# 

**Published:** 1913-09

**Authors:** 


*The following donations have been received since the publication of the list in June.*

*August* 30*th*, 1913.

| | |
|---|---|
| L. M. Griffiths, (1) .. .. .. .. | 1 volume. |
| Dr. H. D. Rolleston (2) .. .. .. .. | 1 ,, |
| Dr. Shingleton Smith (3) .. .. .. | 1 ,, |
| Dr. Francis H. Williams (4) .. .. .. | 1 ,, |

### EIGHTY-NINTH LIST OF BOOKS.

The titles of books mentioned in previous lists are not repeated.

The figures in brackets refer to the figures after the names of the donors, and show by whom the volumes are presented. The books to which no such figures are attached have either been bought from the Library Fund, or received through the *Journal.*

**Bernstein, J. M.** .. *Applied Pathology* .. .. .. .. .. [1913]
**Cunningham, D. J.** *Text-Book of Anatomy* (Edited by A. Robinson) .. .. .. .. .. .. .. .. 4th Ed. 1913
**Dolan, T. M.** .. *Our State Hospitals* .. .. .. .. .. .. .. .. [N.D.]
**Fischer, M. H.** .. *Zur Behandlung der Nephritis* .. .. .. .. 1913
**Haines, H. A.** (Ed.) *Memorial of Charles Morehead* .. .. (1) [1884]
**Henriksen, P. B.** .. *Nervenregeneration* .. .. .. .. .. .. .. .. 1913
**Herman and R. D. Maxwell, G. E.** *Student's Handbook of Gynæcology* 2nd Ed. 1913
**Jones, H. L.** .. *Ionic Medication* .. .. .. .. .. .. .. .. 1913
**Lees, D. B.** .. .. *Incipient Pulmonary Tuberculosis* .. .. .. .. 1913
**Lyth, E. R.** .. .. *Influence of Thermal Environment* .. .. 1913
**Maxwell, G. E. Herman and R. D.** *Student's Handbook of Gynæcology* 2nd Ed. 1913
**Rolleston, H. D.** .. *On Writing Theses* .. .. .. .. .. .. (2) [1911]
**Sawyer, Sir J.** .. *Coprostosis* .. .. .. .. .. .. .. .. .. .. .. 1912
**Walsh, D.** .. .. *Diseases of the Skin* .. .. .. .. .. .. .. 1913
**Wanklyn, W. M.** .. *How to Diagnose Smallpox* .. .. .. .. .. 1913
**Williams, F. H.** .. *The Rœntgen Rays in Medicine* (4) 3rd Ed. 1903
**Wright, D. D'A.** .. *Treatment of Hæmorrhoids* .. .. .. .. .. 1913

### TRANSACTIONS, REPORTS, JOURNALS, &c.

Archiv für klinische Chirurgie .. .. .. .. .. Bd. XCIX 1912
Bäder Almanach .. .. .. .. .. .. .. .. .. .. .. .. .. (3) 1913
British Medical Journal, The .. .. .. .. .. .. .. .. Vol. I 1913
Bulletin de l'Académie royale de Médecine de Belgique Tom. XXVI 1912
Clinical Journal, The .. .. .. .. .. .. .. .. .. .. Vol. XLI 1912–13
Deutsche Zeitschrift für Chirurgie .. .. .. .. .. .. Bd. CXX 1913
Hospitals and Charities, Burdett's .. .. .. .. .. .. .. .. .. 1913
Journal of Hygiene, The .. .. .. .. .. .. .. .. Vol. XII 1912
Lancet, The .. .. .. .. .. .. .. .. .. .. .. .. .. .. .. Vol. I 1913
Library, The .. .. .. .. .. .. .. .. .. .. .. 3rd ser., Vol. III 1912
Luzerne County Medical Society, Transactions of the .. .. 1911–12
Progressive Medicine .. .. .. .. .. .. .. .. .. Vols. I, II 1913
Smithsonian Report for 1911 .. .. .. .. .. .. .. .. .. .. .. 1912
Wiener klinische Wochenschrift .. .. .. .. .. .. .. .. .. .. 1912

### Local Medical Notes.

**University of Bristol.**—Students of the University have recently passed the following examinations :—

University of Bristol.—*M.B., Ch.B.—Intermediate Examination :* Oliver C. M. Davis, H. Archer. *Final Examination, Part II :* E. Christofferson. *M.D.* (*Honours*) *:* R. S. S. Statham. *D.P.H. :* T. S. Bradburn, Christopher C. Court ;

*Part I,* W. R. Cooper, J. R. Kay-Mouat; *Part II,* P. Moxey. *B.D.S.—2nd Examination:* W. H. Greet, R. H. Turner.

F.R.C.S. England.—A. E. Iles.

D.P.H. Lond.—R. G. Johnson.

University of London.—*M.B., B.S.:* Helen P. Barnes, W. Salisbury, E. W. Wade. *1st Examination for Medical Degrees:* R. B. Britton, D. W. Evans, W. H. Royal, G. E. Tilsley. *2nd Examination (Part I):* J. D. Champney, A. H. Morris.

Conjoint Examining Board of England.—*Medicine, Surgery, Midwifery:* H. J. Orr-Ewing.* *Medicine:* W. E. Taylor, H. J. D. Smythe. *Surgery:* H. W. Cooke.* *Midwifery:* H. J. D. Smythe, B. J. Fayle; *2nd Examination,* Margaret Lister.

L.D.S., R.C.S. Eng.—*Chemistry and Physics:* H. C. R. Aubrey, C. M. John, R. V. Knight, W. H. B. Stride, E. F. Vowles. *Chemistry:* G. M. Haydon, G. M. Hicks, W. G. Milton. *Physics:* A. L. Morgan.

### APPOINTMENTS.

George Parker, M.A., M.D., has been appointed Sole Lecturer in Forensic Medicine and Toxicology at University of Bristol.

Oliver C. M. Davis, D.Sc., has been appointed Demonstrator of Forensic Medicine and Toxicology at the University of Bristol.

R. S. Statham, M.D., Ch.B., has been appointed Registrar to Special Departments at Bristol Royal Infirmary.

B. C. Eskell, M.B., Ch.B. Bristol, M.R.C.S., L.R.C.P., has been appointed Honorary Assistant Anæsthetist at the Bristol Royal Infirmary.

G. W. Spencer, B.A., M.B., B.C. Cantab., M.R.C.S., L.R.C.P., has been appointed House Physician at Bristol Royal Infirmary.

R. A. Kerr, M.B., B.Ch., B.A.O. Belfast, has been appointed House Surgeon at Bristol Royal Infirmary.

W. Salisbury, M.B., B.S. Lond., M.R.C.S., L.R.C.P., has been appointed House Surgeon at the Bristol Royal Infirmary.

H. W. Cooke, M.R.C.S., L.R.C.P., has been appointed Resident Obstetric Officer at Bristol Royal Infirmary.

E. W. Wade, M.B., B.S. Lond., has been appointed House Physician to Bristol General Hospital.

A. E. Iles, F.R.C.S. Eng., has been appointed House Surgeon to Bristol Eye Hospital.

C. J. C. Faill, M.R.C.S. Edin., L.R.C.P. Edin., has been appointed Tuberculosis Medical Officer for the City and County of Bristol.

* Denotes qualification.

### *PUBLICATIONS RECEIVED.*

From Baillière, Tindall and Cox, London :
*Diseases of the Skin.* By David Walsh, M.D. 1913. 6s. net.

From John Bale, Sons & Danielsson, Ltd., London :
*Studies on the Influence of Thermal Environment on the Circulation and the Body-Heat.* By Edgar R. Lyth, M.B. 1913. 2s. 6d. net.

From Cassell and Company, Ltd., London :
*The Student's Handbook of Gynæcology.* By George Ernest Herman, M.B. Second Edition, revised by the Author with additions by R. Drummond Maxwell, M.D. 1913. 7s. 6d. net.

From Cornish Bros., Birmingham :
*Coprostasis: its Causes, Prevention and Treatment.* By Sir James Sawyer. 1912. 2s. 6d. net.

From Henry Frowde and Hodder & Stoughton, London :
Cunningham's *Text-Book of Anatomy.* Edited by Arthur Robinson, M.D. Fourth Edition. 1913. 31s. 6d. net.

From Henry J. Glaisher, London :
*The Treatment of Hemorrhoids and Rectal Prolapse by Means of Interstitial Injections.* By Dudley D'A. Wright, F.R.C.S. 1913. 1s. net.

From H. K. Lewis, London :
*Ionic Medication.* By H. Lewis Jones, M.D. 1913. 5s. net.
Lewis's *Pocket Case Book.* 1s. 6d. net.
*The Bradshaw Lecture on Incipient Pulmonary Tuberculosis.* By David Bridge Lees, M.D. 1913. Price, 5s. net.

From Steen'ske Bogtrykkeri, Christiania :
*Nye undersokelser over Nervenregeneration.* Av Dr. Paul B. Henriksen. 1913.

From Theodor Steinkopff, Dresden :
*Weitere Beiträge zur Behandlung der Nephritis und verwandter Erscheinungen.* Von Martin H. Fischer. 1913.

From Smith, Elder & Co., London :
*How to Diagnose Smallpox.* By W. McC. Wanklyn, B.A., D.P.H. 1913. 3s. 6d. net.

From University of London Press, London :
*Applied Pathology.* By Julius M. Bernstein, M.B., D.P.H. [1913.] Price, 10s. 6d. net.

From John Wright & Sons, Ltd., Bristol :
*The British Journal of Surgery.* Vol. I, No. 1, July, 1913. 25s. per annum.

### Bristol Medico-Chirurgical Journal.

**TERMS FOR ADVERTISEMENTS.**

| | £ | s. | d. |
|---|---|---|---|
| Whole Page ... ... ... ... ... ... | 1 | 1 | 0 |
| Half Page ... ... ... ... ... ... ... | 0 | 12 | 6 |
| Quarter Page ... ... ... ... ... ... | 0 | 7 | 6 |

Insets (2 or 4 pages), 20/-

Discount of 5 per cent. will be allowed if payment is made at the time of ordering.

Terms for special positions upon application.

*Orders to be sent to the Publishers,*

J. W. ARROWSMITH LTD., 11 Quay Street, Bristol.

